# Fe-Doped Sol-Gel Glasses and Glass-Ceramics for Magnetic Hyperthermia

**DOI:** 10.3390/ma11010173

**Published:** 2018-01-22

**Authors:** Francesco Baino, Elisa Fiume, Marta Miola, Federica Leone, Barbara Onida, Francesco Laviano, Roberto Gerbaldo, Enrica Verné

**Affiliations:** Department of Applied Science and Technology, Politecnico di Torino, Corso Duca degli Abruzzi 24, 10129 Torino, Italy; elisa.fiume@polito.it (E.F.); marta.miola@polito.it (M.M.); federica.leone@polito.it (F.L.); barbara.onida@polito.it (B.O.); francesco.laviano@polito.it (F.L.); roberto.gerbaldo@polito.it (R.G.)

**Keywords:** bioactive glass, sol-gel, thermal properties, magnetic, magnetite, mesoporous, hyperthermia, cancer treatment

## Abstract

This work deals with the synthesis and characterization of novel Fe-containing sol-gel materials obtained by modifying the composition of a binary SiO_2_-CaO parent glass with the addition of Fe_2_O_3_. The effect of different processing conditions (calcination in air vs. argon flowing) on the formation of magnetic crystalline phases was investigated. The produced materials were analyzed from thermal (hot-stage microscopy, differential thermal analysis, and differential thermal calorimetry) and microstructural (X-ray diffraction) viewpoints to assess both the behavior upon heating and the development of crystalline phases. N_2_ adsorption–desorption measurements allowed determining that these materials have high surface area (40–120 m^2^/g) and mesoporous texture with mesopore size in the range of 18 to 30 nm. It was assessed that the magnetic properties can actually be tailored by controlling the Fe content and the environmental conditions (oxidant vs. inert atmosphere) during calcination. The glasses and glass-ceramics developed in this work show promise for applications in bone tissue healing which require the use of biocompatible magnetic implants able to elicit therapeutic actions, such as hyperthermia for bone cancer treatment.

## 1. Introduction

Magnetic materials represent advanced solutions suitable for a wide range of biomedical applications, according to their different magnetic responses to an applied external magnetic field [1]. Specific applications of these materials include magnetic resonance imaging (MRI), magnetic stimulation, magnetic cell separation, active targeting for drug delivery applications, protein immobilization and hyperthermia for the treatment of malignant tumors [2,3,4].

The most commonly used magnetic materials in biomedical applications are ferrimagnetic iron oxides (e.g., magnetite, Fe_3_O_4_), although their acute toxicity and their still partially unknown fate in vivo limit their usage as implants in clinical practice [5]. These drawbacks can be partially overcome by embedding the magnetic phase within a biocompatible matrix (e.g., glasses or ceramics) [6,7,8,9].

Magnetic induction of hyperthermia is based on the use of implanted magnetic materials to generate heat in the site of concern under the application of an external magnetic field [10]. In a typical magnetic hyperthermia treatment, heat is generated by applying an alternating magnetic field to the magnetic materials implanted or injected into the tumor site in order to increase and maintain the temperature in the neoplastic tissue around 41–45 °C. Malignant cells are selectively killed while being exposed to such temperatures because heat is slowly dissipated in cancerous tissues due to the lack of a well-organized vascular network; moreover, hyperthermia causes many changes in cells and leads to a loss of cellular homeostasis [11].

Biocompatible magnetic glasses have recently attracted the attention of several research groups for possible use in the advanced treatment of bone cancer as a complementary approach to chemotherapy, which is known to carry many side effects to patients. Fe_3_O_4_-containing melt-derived glass-ceramics were extensively investigated in a series of studies by Verné and coworkers and were found highly promising for the treatment of osseous tumors by hyperthermia [12,13,14]. Interestingly, a pro-osteogenic activity was observed in an acrylic-based composite bone cement containing 10% of ferrimagnetic glass-ceramic, with a synergistic effect between bioactivity and cell mineralization [12].

Apart from the classical melting-quenching route, sol-gel method has also been applied to produce magnetic glasses. Shankhwar et al. [15] synthesized sol-gel 45S5-based bioactive glasses containing iron oxide and reported that a thermal treatment at 850 °C led to the formation of sodium calcium silicate and magnetic phases into the glassy matrix.

Ferreira da Silva et al. [16] prepared sol-gel ZnO-Fe_2_O_3_-SiO_2_ glasses containing zinc ferrite nanoparticles that, after being treated at 500 °C, exhibited ferro- or ferrimagnetic interactions combined with superparamagnetism with a blocking temperature of −259 °C.

Coroiu et al. [17] studied the magnetic and structural behavior of a sol-gel derived ternary composition containing both iron and aluminum. The results indicated a correlation between the heat-treatment temperature and the nucleation of specific crystalline phases, since hematite (α-Fe_2_O_3_) was observed resulting from the complete conversion of goethite (FeO(OH)) by increasing the temperature up to 500 °C. Hypothesis of the modification of the internal network of the samples was supported by infrared spectroscopy (IR) data, as a result of the conversion of Fe^2+^ into Fe^3+^ ions.

An alternative to the introduction of iron oxides during the sol-gel process was proposed by Baikousi et al. [18], who produced CaO-SiO_2_-P_2_O_5_ ternary bioactive glasses functionalized with magnetic nanoparticles. Specifically, nanocomposite materials were synthesized with both bioactive and magnetic properties by homogeneously dispersing magnetic nanoparticles in a glassy matrix. The base glass was produced by traditional sol-gel route and then glass powders were dispersed within a methanol solution containing iron nitrate tetra-hydrate; after stirring and removal of the solvent, the obtained solid precipitate was powdered, exposed to vapors of acetic acid and dried. Calcination of the glass was performed at different temperatures under argon flowing atmosphere. Results showed that magnetite phases (magnetite and maghemite) were homogeneously dispersed within the porous structure and the glass matrix had high stability towards crystallization at high temperatures (up to 800 °C), regardless the iron content. 

Bioactive glasses are known to be osteoinductive materials as their ionic dissolution products stimulate the cell genes toward a path of regeneration and self-repair [19]. Depending on both composition (e.g., types of network-forming oxides and modifiers used) and textural features (i.e., non-porous, macroporous or mesoporous glasses), the glass reactivity can be modulated so that various degradation rates and apatite-forming kinetics can be properly designed [20,21]. Small amounts of metallic ions, such as B^3+^ [22], Cu^2+^ [23], Ag^+^ [24], Sr^2+^ [25] and Co^2+^ [26], have also been used as dopants for bioactive glasses to elicit additional therapeutic functions (e.g., angiogenesis and antiseptic effect). Interestingly, incorporation of Fe within a mesoporous silicate bioactive glass was reported to enhance mitochondrial activity and expression of osteogenesis-related genes (ALP and OCN) in human bone marrow mesenchymal stem cells, thus supporting the hypothesis of the great potential of such materials in clinical applications concerning bone healing (repair of large bone defects caused by malignant bone tumors through a combination of osteo-conductive properties and hyperthermic approach) [27]. The fascinating perspective of directing tissue regeneration by magnetic activation was also explored by Russo et al. [28], who observed enhanced bone regeneration in vivo (rabbit) when magnetized F_3_O_4_-containing hydroxyapatite/collagen composite scaffolds were used as compared to non-magnetic control.

In the present work, sol-gel glasses and glass-ceramics in the SiO_2_-CaO-F_2_O_3_ system were produced by sol-gel method and the effects of heat-treatment conditions on thermal behavior, crystallization and magnetic properties were investigated.

## 2. Materials and methods

### 2.1. Preparation of Materials

Materials were produced by a sol-gel route; the oxide compositions of the three systems are reported in Table 1. Two novel Fe-containing ternary glasses, 60S38C2Fe and 60S30C10Fe, were obtained by modifying the 60S40C Fe-free binary composition, which was used as a control system for the analysis of results. Tetraethyl orthosilicate (TEOS), calcium nitrate tetrahydrate (Ca(NO_3_)_2_·4H_2_O (CaNT)) and iron chloride (FeCl_3_) (all purchased from Sigma-Aldrich, St. Louis, MO, USA) were used as SiO_2_, CaO and Fe_2_O_3_ sources, respectively. In a typical synthesis, 1.2 mL of 2 N HNO_3_ were added to 7.2 mL of distilled water and the solution was mixed in sealed flasks for 5 min; afterwards, TEOS and CaNT were added under continuous magnetic stirring (200 rpm) in 2 h intervals. The molar ratio (H_2_O + HNO_3_) to TEOS was about 8.0 in all the syntheses.

The sol prepared for the synthesis of the control system (60S40C) was ready to undergo gelation; however, an additional step was required for the synthesis of the Fe-containing materials. Specifically, FeCl_3_ was slowly added to the batch which was stirred for 1 h until complete dissolution of the salt. 

Gelation of the sols was carried out at room temperature in sealed flasks for 3 days, followed by ageing at 60 °C for 72 h. Slow solvent evaporation was then allowed by opening the containers and increasing the temperature up to 140 °C for 48 h. At the end of this thermal treatment, the dried gels appeared cracked because of the internal stresses resulting from the drying process. These samples were milled (single-ball zirconia milling machine, Pulverisette 0, Fritsch, Idar-Oberstein, Germany), labelled as “Sample code-140”, and stored for subsequent analyses. Dried gels were thermally stabilized (calcined) at 700 °C for 3 h (heating rate 1 °C/min) in air or inert atmosphere (argon (Ar) flow) to allow densification of the matrix, ball-milled and labelled as “Sample code-air” or “Sample code-Ar” depending on the treatment conditions. High-temperature thermal treatment under argon flowing (non-oxidant conditions) was thought as a potential strategy to promote the development of magnetite in the Fe-doped materials. Calcination was performed at 700 °C for all samples as this temperature is enough to induce the nucleation of magnetite [29] and a comparison, especially in terms of magnetic properties, with Fe-doped mesoporous glasses produced elsewhere is allowed [27].

**Figure 1:**
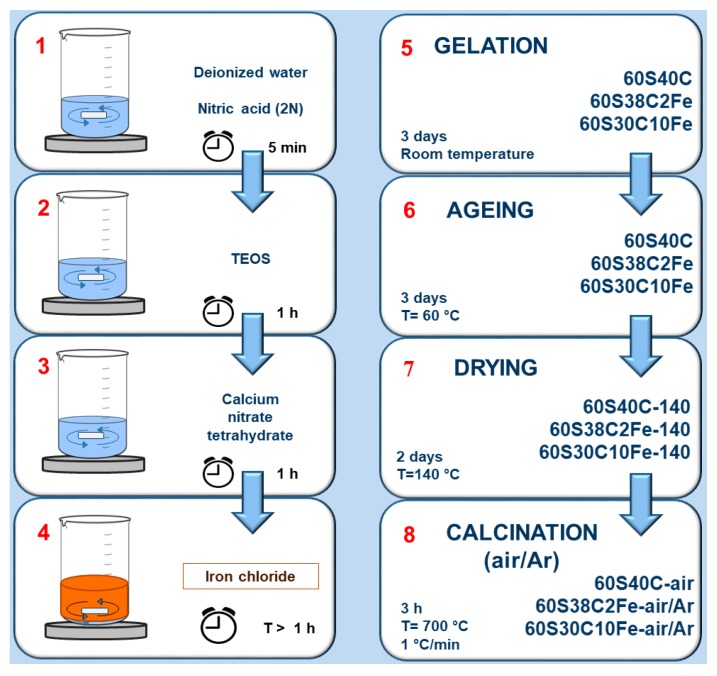
Scheme of the eight-stage synthesis process adopted to produce the materials investigated in this work.

The workflow reported in Figure 1 summarizes the main phases of the synthesis process and the materials obtained.

### 2.2. Characterization

#### 2.2.1. Microstructural Analysis

Powdered materials underwent wide-angle X-ray diffraction (XRD; 2θ within 10–70°) by using a X’Pert Pro PW3040/60 diffractometer (PANalytical, Eindhoven, The Netherlands) operating at 40 kV and 30 mA with Bragg–Brentano camera geometry, Cu Kα incident radiation (wavelength λ = 0.15405 nm), step size Δ(2θ) = 0.02° and fixed counting time of 1 s per step. Identification of crystalline phases was performed by using X’Pert HighScore software (2.2b) equipped with the PCPDFWIN database (http://pcpdfwin.updatestar.com). 

#### 2.2.2. Thermal Analyses

Thermal analyses were carried out on both calcined and non-calcined materials, i.e., at both Step 8 and Step 7 of the production process, as reported in the workflow of Figure 1. 

Differential thermal analysis (DTA) was performed on powdered calcined samples (50 mg) by using a DTA 404 PC instrument (Netzsch, Selb, Germany); temperature range was 20–1400 °C with a heating rate of 10 °C/min. The powder was introduced in Al_2_O_3_ crucibles provided by the manufacturer; high-purity Al_2_O_3_ powder was used as a reference material. Standard calibration procedure and baseline corrections were performed.

Calcined samples also underwent hot-stage microscopy (HSM) by making use of a hot-stage instrument equipped with electrical furnace and image analysis software (Expert System Solution, Modena, Italy). Specimens of pressed powder (diameter ~1 mm, height ~3 mm) were positioned onto a high-purity Al_2_O_3_ plate; then, black-and-white images (silhouettes) of the sample profile were taken from 25 to 1450 °C with a heating rate of 10 °C/min. The variation of the sample dimensions upon heating were measured and the shrinkage (%) was quantified, along with the temperature of first shrinkage (T_FS_) and maximum shrinkage (T_MS_).

DTA and HSM measurements were carried out in either air (oxidizing atmosphere) or argon flow (inert atmosphere), consistently to the treatment conditions of the samples during calcination.

Non-calcined samples (“Sample code-140” set) were investigated through differential scanning calorimetry (DSC) by using a DSC 404 F3 Pegasus^®^ instrument (Netzsch, Selb, Germany); measurement conditions were analogous to those adopted for DTA on calcined samples.

The characteristic temperatures of the materials, i.e., glass transition temperature (T_g_), onset of crystallization (T_x_), crystallization temperature (T_c_) and melting temperature (T_m_), were estimated directly from the DTA or DSC plots.

#### 2.2.3. Morphology, Composition and Porosity

Thermally-stabilized sol-gel materials were investigated by field-emission scanning electron microscopy (FESEM; Supra^TM^ 40, Zeiss, Oberkochen, Germany) to evaluate particle size and shape. The samples were sputter-coated with chromium prior to the analysis and inspected at an accelerating voltage of 15 kV. Compositional investigations were also performed by energy dispersive spectroscopy (EDS), which was included in the FESEM equipment.

Nitrogen (N_2_) adsorption-desorption porosimetry measurements were performed at −196 °C (Quantachrome Autosorb1, Quantachrome, Boynton Beach, FL, USA) on the materials calcined in air and argon. The specific surface area (SSA) was assessed by using the Brunauer-Emmet-Teller (BET) method [30], and the pore size distribution (along with the mean pore size) was determined by the non-local density functional (NLDFT) theory approach [31].

#### 2.2.4. Magnetic Properties

DC magnetic properties were investigated by means of a DC magnetometer/AC susceptometer (LakeShore 7225) equipped with a Cryogen-Free Magnet system (LakeShore Cryotronics, Westerville, OH, USA) at room temperature in quasi-static condition. In particular, magnetic hysteresis cycle measurements up to 1600 kA/m were performed on the samples calcined at 700 °C in both air and argon to estimate the main magnetic parameters of the materials (i.e., remanent magnetization, coercitive force, saturation magnetization, and magnetic hysteresis loss calculated from the area of hysteresis loop).

## 3. Results and Discussion

**Figure 2:**
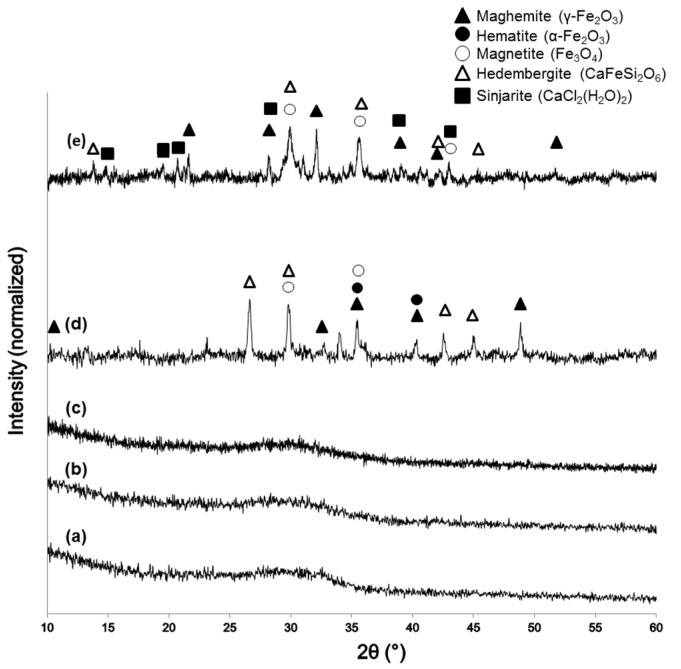
XRD patters of: (**a**) 60S40C-air; (**b**) 60S38C2Fe-air; (**c**) 60S38C2Fe-Ar; (**d**) 60S30C10Fe-air; and (**e**) 60S30C10Fe-Ar.

XRD patterns of the sol-gel materials after calcination are reported in Figure 2. The glassy nature of 60S40C (Figure 2a) and 60S38C2Fe systems (Figure 2b,c) is demonstrated by the presence of a typical broad halo in the 2θ range of 25°–35° without any diffraction peak. The calcination process performed on 60S38C2Fe at 700 °C in either air or argon did not induce the nucleation of any crystalline phases within the glassy matrix, suggesting that the environmental conditions of heat treatment play a secondary role in the devitrification process for this material. No Fe-containing crystalline phases were detected in 60S38C2Fe materials as the majority of iron content was taken up in the glass network. On the contrary, as far as 60S30C10Fe system is concerned, a higher iron content played a key role in promoting the nucleation of different crystalline phases depending on the environmental conditions under which the thermal treatment was performed (Figure 2d,e). As a result, both 60S30C10Fe-air and 60S30C10Fe-Ar are glass-ceramic materials that include Fe-containing crystalline phases. The role played by Fe_2_O_3_ in promoting devitrification of silicate ternary glasses was deeply investigated by Poirier et al. [32], who confirmed its greater effectiveness as devitrifying agent compared to other metal oxides (e.g., TiO_2_). As assessed by the magnetic measurements that will be described later, 60S30C10Fe-Ar exhibited a clear magnetic nature. Hence, it is reasoned that ferrimagnetic maghemite (γ-Fe_2_O_3_) or magnetite (Fe_3_O_4_) exists embedded in the glassy matrix (as actually reported in Figure 2d,e) and are responsible of the observed magnetization. Although distinguishing these two iron oxide crystalline phases is difficult due to the close proximity of their diffraction peak positions, it is likely that maghemite was reduced into magnetite under the heating treatment of calcination in argon, as the former has a lower thermal stability [33]. In this regard, it has been shown that heat treatment of melt-derived glasses containing Fe_2_O_3_ results in the formation of glass-ceramics with Fe_3_O_4_ [34]. The heating treatment in air promotes the conversion of magnetite to hematite (α-Fe_2_O_3_) by oxidation (Figure 2d); these observations are in agreement with previous results reported by other authors on magnetite oxidation [35].

It is interesting to note the presence of a Cl-containing crystalline phase, sinjarite, in the diffraction spectrum of 60S30C10Fe-Ar. The presence of sinjarite was unexpected—no Cl-containing species were included in the nominal composition of the material (see Table 1)—and derived from the reaction between residual Cl^−^ ions (from FeCl_3_ used as F_2_O_3_ precursor during the synthesis process) and Ca^2+^. Although chlorine is often associated to adverse effects on human health (e.g., irritation of skin, eyes and respiratory system), CaCl_2_-derived materials are biocompatible at low dosage and were even suggested as adjuvants for bone tissue engineering applications [36]. Use of alternative precursors for Fe, such as iron nitrates, could be considered in the future to obtain high-purity sol-gel materials.

**Figure 3:**
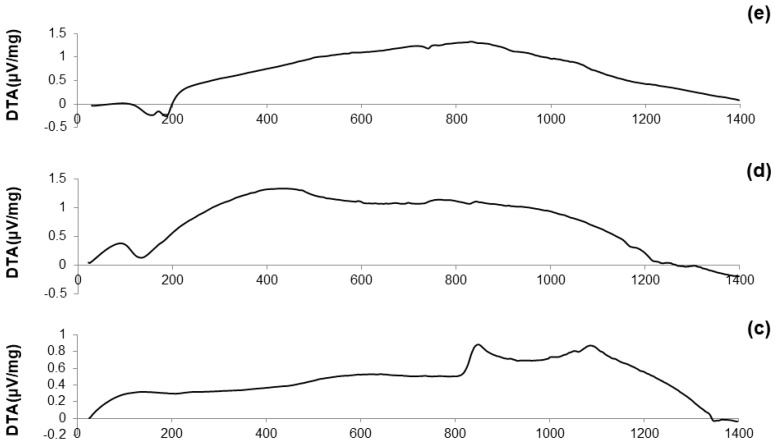

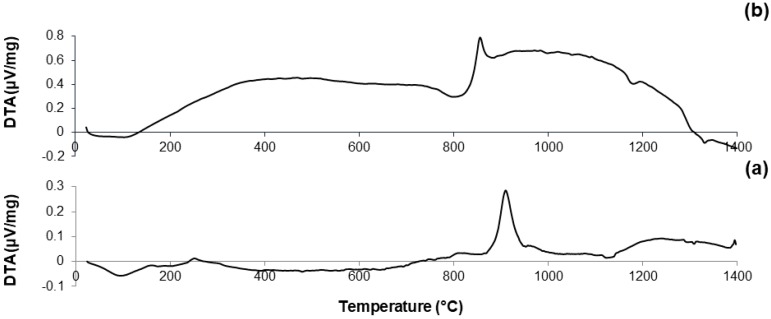
DTA plots of: (**a**) 60S40C-air (measurement performed in air); (**b**) 60S38C2Fe-air (measurement performed in air); (**c**) 60S38C2Fe-Ar (measurement performed in argon); (**d**) 60S30C10Fe-air (measurement performed in air); and (**e**) 60S30C10Fe-Ar (measurement performed in argon).

DTA plots of calcined materials are reported in Figure 3; the characteristic temperatures of the materials analyzed are collected in Table 2. 

Considering the oxide composition of 60S40C, it was reasonable to associate the exothermic crystallization peak (T_x_ = 850 °C, see Figure 3a) to the development of wollastonite (CaSiO_3_) crystals. This hypothesis was confirmed by XRD analysis (not reported here) performed on 60S40C powders that were heat-treated at 850 °C. These results are in accordance with Pérez et al. [37] who found a devitrification experimental range of wollastonite between 850 and 950 °C.

Figure 3b shows that, as the temperature rises above T_c_, the curve evolves in a sort of “hump” until melting point is reached. Two different endothermic peaks associated to melting phenomena were identified, which were related to two different crystalline phases developing upon the thermal cycle. This hypothesis can also explain the “hump” observed in the curve, suggesting the slow development of one (or even more) additional crystalline phase within the system above 900 °C.

The hypothesis about the formation of a second crystalline phase was supported by observing the trend of the DTA curve reported in Figure 3c. The thermograph of 60S38C2Fe-Ar shows two well-distinguishable exothermic peaks of comparable intensity, thus suggesting the importance of the treatment conditions (argon flow vs. air) in affecting the development of an additional crystalline phase. Consistently to what was assumed during the analysis of the DTA curve for 60S40C powders, XRD analysis (not reported here) revealed the presence of wollastonite crystals in the 60S38C2Fe powders that were heat-treated at 800 °C (onset of crystallization, T_x_), thus allowing the attribution of the first exothermic peak to the development of such calcium-silicate phase. 

Figure 3d,e reports the DTA curves related to the calcined 60S30C10Fe system. There are marked differences with respect to the thermographs of the other two compositions, 60S40C and 60S38C2Fe, as neither evident inflection points nor exothermic peaks can be observed; only a melting temperature at about 1180 °C was detected in Figure 3d. The non-amorphous nature of the materials is suggested due to the large amount of iron in the composition, which is responsible for the initiation of devitrification phenomena below the calcination temperature (700 °C); this is in accordance with previous results by other authors [38]. 

HSM was performed on each one of the calcined systems previously analyzed by DTA by subjecting the samples to the same heating cycle, in order to complement the results given by the DTA with further data about the behavior of these materials under thermal treatment. Data from HSM are collected in Table 2.

**Figure 4:**
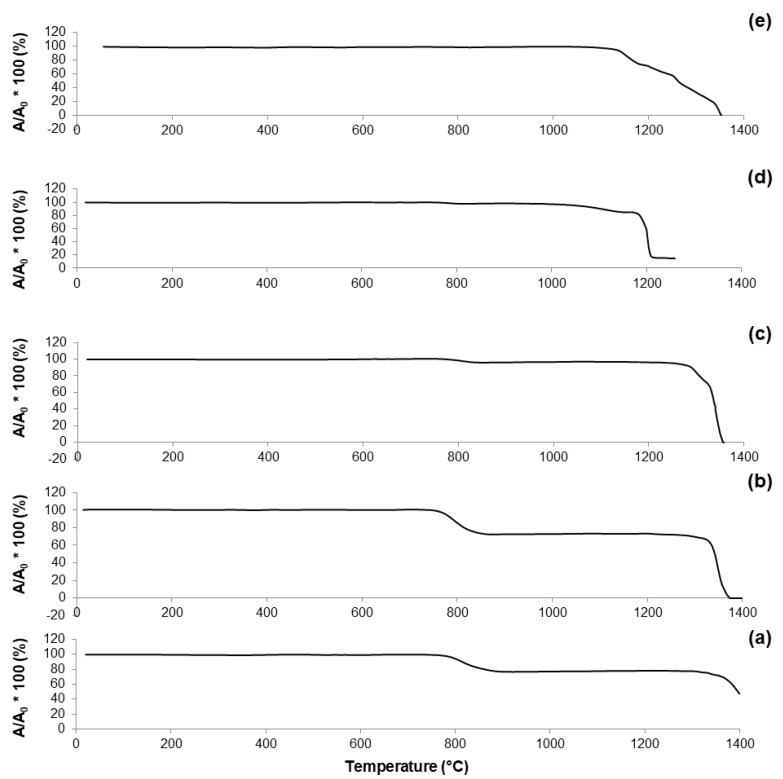
HSM plots (shrinkage variations) of: (**a**) 60S40C-air (measurement performed in air); (**b**) 60S38C2Fe-air (measurement performed in air); (**c**) 60S38C2Fe-Ar (measurement performed in argon); (**d**) 60S30C10Fe-air (measurement performed in air); and (**e**) 60S30C10Fe-Ar (measurement performed in argon).

The curve reported in Figure 4a reveals that 60S40C-air is characterized by a one-stage shrinkage upon heating. This means that the height of the sample does not vary until the shrinkage starts; then, densification occurs followed by a plateau until the sample begins to melt. T_FS_ was identified at 762 °C; as the temperature rises above T_FS_, the curve exhibits a negative slope until maximum shrinkage (T_MS_). A reduction of about 20% in height was observed during the heating treatment of 60S40C-air.

A one-stage shrinkage upon heating was also observed for 60S38C2Fe system (Figure 4b,c). However, even if the trend of both curves is comparable, the reduction in height of the sample (i.e., densification) appears considerably more pronounced in Figure 4b (shrinkage about 30%) compared to Figure 4c (shrinkage about 10%). A clear step at about 1340 °C can be seen in Figure 4c, which can be related to the endothermic peak at 1349 °C observed in the DTA plot (Figure 2c) and associated with melting processes. 

As already observed in the case of DTA plots, calcined 60S30C10Fe shows a significantly different thermal behavior under HSM (Figure 4d,e) compared to the other two systems. Densification of the material occurs following a multi-stage shrinkage process, which does not reflect the typical behavior of a glassy material [39] but supports the hypothesis of the semi-crystalline nature of calcined 60S30C10Fe powders, as actually revealed by XRD investigations (Figure 2d,e). 

**Figure 5:**
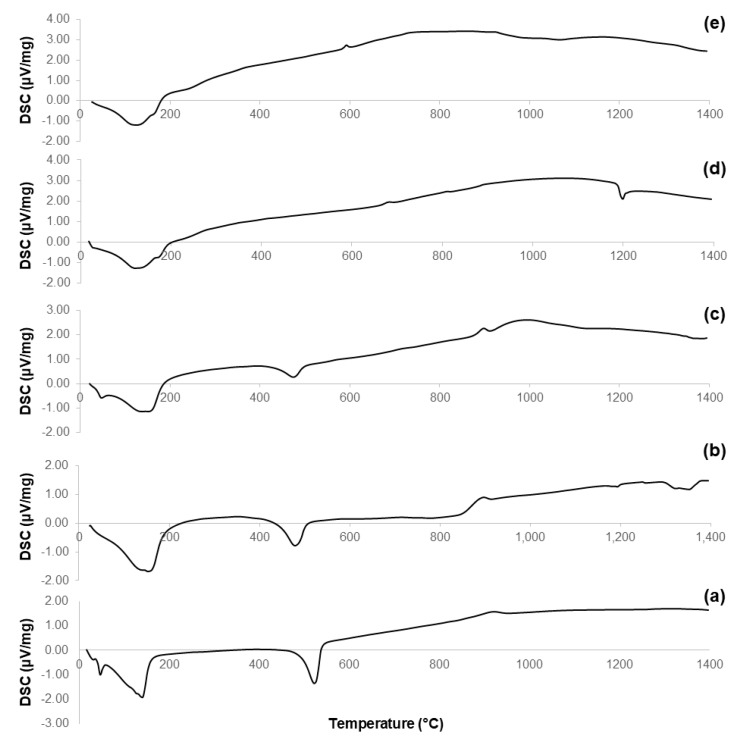
DSC plots of: (**a**) 60S40C-140 carried out in air; (**b**) 60S38C2Fe-140 carried out in air; (**c**) 60S38C2Fe-140 carried out in argon; (**d**) 60S30C10Fe-140 carried out in air; and (**e**) 60S30C10Fe-140 carried out in argon.

Figure 5 reports the results of DSC analyses performed on the non-calcined materials obtained at Step 7 of the synthesis process (see Figure 1). These thermographs allow a better understanding of the evolution of the crystalline phases that appear in the calcined samples (Figure 2); the characteristic temperatures are collected in Table 3. 

The curves associated to 60S40C-140 (Figure 5a) and 60S38C2Fe-140 materials (Figure 5b,c) show an exothermic peak with T_x_ within 840–870 °C, which is consistent with the results obtained by DTA in Figure 3a–c. Hence, it is further confirmed that, if these materials are calcined at 700 °C, no crystalline phases are expected to appear (as actually assessed by XRD, see Figure 2a–c). On the contrary, a clear exothermic signal below 700 °C is visible in the plots of 60S30C10Fe-140 materials (Figure 5d,e). This is consistent with XRD results that reveal the presence of Fe-bearing crystalline phases in these materials after calcination (Figure 2d,e). The onset of crystallization of magnetite for 60S30C10Fe-140 treated in argon (570 °C) is close to the value (550 °C) obtained by other authors who performed DSC analyses on Fe_2_O_3_-SiO_2_-based glasses under argon flowing [40]. 

**Figure 6:**
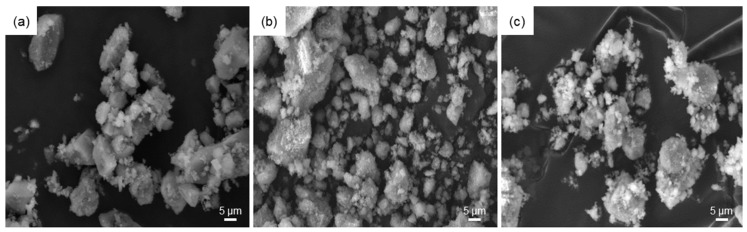
SEM micrographs of: (**a**) 60S40C-air; (**b**) 60S38C2Fe-air; and (**c**) 60S30C10Fe-air powders.

Morphological investigations by SEM (Figure 6) revealed that the particles of all three systems, after being calcined at 700 °C, tend to form aggregates with the larger particles (dimensions of a few tens of micrometers) coated by the finest ones (micrometric or sub-micrometric size), suggesting a bimodal distribution of the particle size. Elemental assessment of materials composition by EDS was in good agreement with the nominal values; acceptable discrepancies between experimental and theoretical Si/Ca molar ratios were recorded (1.53 vs. 1.50 for 60S40C, 1.32 vs. 1.57 for 60S38C2Fe, and 1.90 vs. 2.0 for 60S30C10Fe calcined system). The presence of Cl in the calcined 60S30C10Fe systems was confirmed by EDS, in agreement with the results of XRD analysis. Similar results, in terms of morphology and composition of the powders, were obtained for both air- and Ar-treated samples.

**Figure 7:**
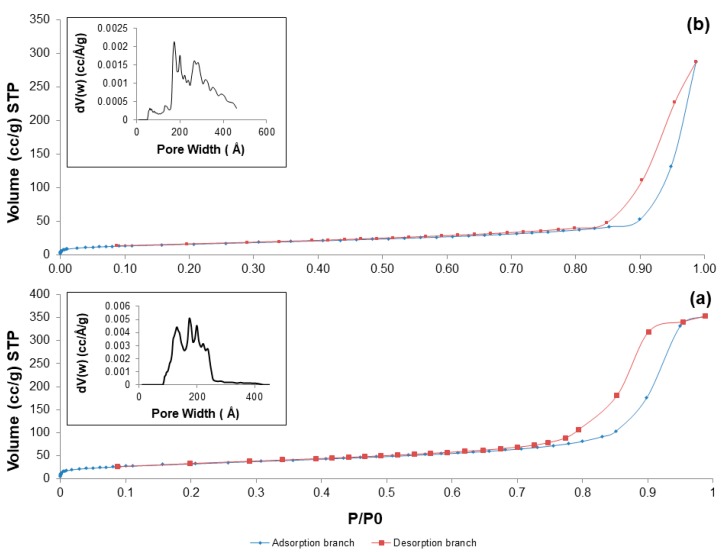
Nitrogen adsorption-esorption isotherms at −196 °C of: (**a**) 60S40C-air; and (**b**) 60S38C2Fe-air. Inset in both pictures reports the pore size distribution assessed by NLDFT method.

Nitrogen adsorption-desorption measurements confirmed the mesoporous structure of the produced materials after calcination at 700 °C in both air and argon: in fact, all the five systems exhibited a type-IV isotherm pattern (Figure 7), typical of mesoporous materials [41]. According to the IUPAC definition, mesoporous materials are characterized by pore size in the range of 2 to 50 nm [42].

As reported elsewhere [43], the shape of the hysteresis loop is closely related to the shape of mesopores. 60S40C-air exhibits a H2 hysteresis loop, corresponding to mesopores with undefined shape, while the H3 hysteresis loop of Fe-containing systems suggests the presence of slit-shaped pores. Textural parameters are summarized in Table 4. 

60S40C-air exhibits a SSA comparable to the value reported by Sepuveda et al. for 58S sol-gel glass of commercial origin (around 125–165 m^2^/g) [44]. The data collected in Table 4 suggest that addition of iron to the base glass composition involves a decrease of SSA, which undergoes a further decrement if calcination is performed in inert atmosphere. Thermal treatment under argon flowing also induces a decrease of the mean pores size that reaches the typical values exhibited by mesoporous silica (e.g., MCM-41) and mesoporous bioactive glasses (below 10 nm). This is an interesting finding that suggests a new strategy for modulating the textural parameters of sol-gel materials by acting on the calcination environmental conditions. The suitability of 60S38C2Fe-Ar and 60S30C10Fe-Ar as drug delivery vehicles could also deserve to be investigated in a future work, since mesoporous materials having pore size comparable to the dimensions of many therapeutic biomolecules (few nanometers) show great promise as smart platforms for the controlled release of drugs and growth factors [45].

A robust comparison between the textural data reported in Table 3 and the previous literature is not possible due to the relative pbucity of publications about Fe-doped sol-gel biomedical glasses. Shankhwar and Srinivasan [15] reported the synthesis of magnetic 45S5 sol-gel bioactive glasses without investigating the textural characteristics. According to Wu et al. [27], the SSA of mesoporous glasses doped with 10 mol % of Fe was around 268 m^2^/g, but this material was synthesized by using a surfactant (Pluronic P123) as a template for the mesoporous structure, which is not carried out in conventional sol-gel processes and leads to higher pore volumes. In general, the SSA of 60S40C, 60S38C2Fe and 60S30C10Fe systems is consistent with the values that are typically obtained for sol-gel materials (a few tens of m^2^/g) [46] and significantly higher than the SSA of melt-derived glasses (around 0.10 m^2^/g) [47]. 

**Figure 8:**
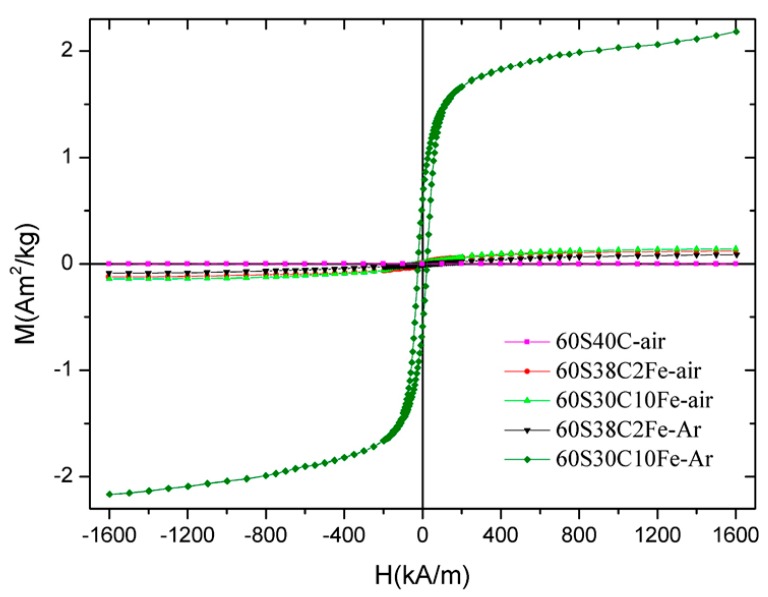
Magnetization curves (hysteresis cycles) of the calcined materials up to 1600 kA/m.

The hysteresis cycles up to 1600 kA/m for all the five samples calcined at 700 °C are collectively shown in Figure 8; the magnetic parameters are reported in Table 5. 

A significant difference can be noted between the values obtained for 60S30C10Fe-Ar and those associated to the other samples. This confirms the presence of ferrous phases with higher magnetic signal (magnetite or maghemite, as assessed by XRD measurements). In particular, the magnetization value at 1600 kA/m for 60S30C10Fe-Ar is 20–25 times that of 60S38C2Fe-air/60S38C2Fe-Ar and 15 times that of 60S30C10Fe-air. The magnetic parameters of 60S30C10Fe-Ar are comparable to those reported by other authors for a magnetite-containing sol-gel glass with a 45S5 basic composition [15]. Interestingly, the saturation magnetization of 60S30C10Fe-Ar is significantly higher than the value (1.32 Am^2^/kg reported for a SiO_2_-CaO-P_2_O_5_ mesoporous bioactive glass (MBG) doped with 10 mol % of Fe and calcined at 700 °C in argon [48], as done in the present work. Aqueous suspensions of this Fe-doped MBG were exposed to an alternating magnetic field and it was reported that the material could effectively generate heat to raise the temperature of the surrounding environment (from 37 to 44.5 °C after 20 min) [48]. As a direct comparison is possible between the two materials, it is expected that 60S30C10Fe-Ar may be suitable for applications in magnetic hyperthermia, too. 

The lower magnetization of 60S30C10Fe-air is due to the predominant presence of hematite (Figure 8d), which is antiferromagnetic [49]. Interestingly, the amorphous 60S38C2Fe materials produce a magnetic response, too, albeit a poor one compared to the other materials. This micromagnetic behavior reflects the presence of long-range (ferri)magnetic interactions among the iron ions that are present in the glassy matrix [50]. In this regard, it has been shown that two types of interactions exist in glassy systems containing Fe_2_O_3_ as network modifiers, i.e., dipole-dipole and superexchange-type interactions [51]. A similar weak magnetic response was also reported by Wu et al. [27] for SiO_2_-CaO-P_2_O_5_ mesoporous glass scaffolds doped with 5 mol % of F_2_O_3_, but the magnetic parameters were not quantitatively assessed. 

In vitro biocompatibility of 60S38C2Fe and 60S30C10Fe with appropriate bone cells remains to be studied and will deserve to be the topic of future research. Previous investigations on these biological aspects performed by other authors using similar biomaterials show great promise. It was reported that doping of SiO_2_-CaO-P_2_O_5_ mesoporous sol-gel glasses with 5–10 mol % of F_2_O_3_ does not suppress the apatite-forming ability of the material [27], which is key to promote healthy osteoblast growth and differentiation as well as bone-bonding and osteogenesis in vivo [52]. Furthermore, glasses containing small amounts of Fe were found to improve the viability of bone marrow mesenchymal stem cells (BMSCs) and the expression of bone-related genes compared to Fe-free glass, which suggest the non-toxicity of these Fe-doped materials coupled to enhanced bone-regenerative properties [27]. 

## 4. Conclusions

Different amounts of Fe_2_O_3_ (2 and 10 mol %) were introduced into the composition of a sol-gel silicate glass with the aim of imparting magnetic properties to the materials for possible use in the treatment of malignant bone tumors by hyperthermia. Samples with composition 60S40C and 60S38C2Fe were characterized by an amorphous structure, regardless of the thermal treatment performed (air vs. argon flowing). Low amounts of Fe_2_O_3_ within the glass composition did not induce devitrification of the system during calcination at 700 °C. The addition of 10 mol % of Fe_2_O_3_ led to the formation of a glass-ceramic material, the crystalline phases of which were highly dependent on the thermal treatment performed: in fact, while the formation of hematite was promoted in oxidant atmosphere (air), the presence of ferrimagnetic phases (maghemite/magnetite) was assessed in the glassy matrix if calcination was performed under argon flowing. All calcined materials exhibited high surface area and mesoporous texture typical of glasses/glass-ceramic produced by sol-gel method. Based on these promising results, further research will focus on the bioactivity of these materials to assess if they are suitable for use as multifunctional implants able to simultaneously promote bone regeneration and elicit a local hyperthermic effect, which are key properties for the successful treatment of bone cancer.

## Figures and Tables

**Table 1:** Nominal compositions (mol %) and reactants of the sol-gel materials produced in this work ^a^.

Sample Code	Composition (mol %)	TEOS (mL)	CaNT (g)	FeCl_3_ (g)
**60S40C**	60SiO_2_-40CaO	11.66	8.22	-
**60S38C2Fe**	60SiO_2_-38CaO-2Fe_2_O_3_	11.66	7.81	0.56
**60S30C10Fe**	60SiO_2_-30CaO-10Fe_2_O_3_	11.66	6.17	2.82

^a^ All the syntheses were carried out with 7.2 mL of distilled water and 1.2 mL of 2 N HNO_3_ to obtain 20 mL of sol.

**Table 2:** Characteristic temperatures of the calcined materials determined (when possible) from DTA (T_g_, T_x_, T_c_ and T_m_) and HSM plots (T_FS_ and T_MS_).

Sample	T_g_ (°C)	T_x_ (°C)	T_c_ (°C)	T_m_ (°C)	T_FS_ (°C)	T_MS_ (°C)
60S40C-air	700	850	910	1384	762	879
60S38C2Fe-air	735	800	855	1180, 1330	747	864
60S38C2Fe-Ar	680	800	847, 1087	1195, 1349	765	843
60S30C10Fe-air	-	-	-	1180	759	999
60S30C10Fe-Ar	-	-	-	-	1062	1158

**Table 3:** Characteristic temperatures of non-calcined materials determined (when possible) from DSC plots.

Sample	T_g_ (°C)	T_x_ (°C)	T_c_ (°C)
60S40C-140 in air	700	840	920
60S38C2Fe-140 in air	690	850	900
60S38C2Fe-140 in argon	697	870	900, 990
60S30C10Fe-140 in air	-	660	680, 807, 900
60S30C10Fe-140 in argon	-	570	590, 740, 920

**Table 4:** Textural parameters obtained by N_2_ adsorption–desorption porosimetry for the calcined sol-gel materials.

Sample	SSA (m^2^/g)	D_NLDFT_ (nm)
60S40C-air	119.4	18.4
60S38C2Fe-air	59.7	29.8
60S38C2Fe-Ar	7.4	6.1
60S30C10Fe-air	41.5	26.4
60S30C10Fe-Ar	11.7	4.9

**Table 5:** Magnetic parameters evaluated from hysteresis cycles of the calcined materials.

Sample	Remanent Magnetization (Am^2^/kg)	Coercitive Force (kA/m)	Saturation Magnetization (Am^2^/kg)	Hysteresis Area at ±1600 kA/m (J/kg)
60S30C10Fe-Ar	0.6	22	2.17	0.16
60S30C10Fe-air	0.03	2.5	0.14	0.02
60S38C2Fe-Ar	<0.02	2	0.09	<0.01
60S38C2Fe-air	<0.02	2	0.11	<0.01
